# Quarantine Convention

**Published:** 1858-03

**Authors:** 


QUARANTINE CONVENTION.—The Commissioners appointed by the Quarantine Convention, which was held in this city last May, met in the Hall of the College of Physicians, on Thursday afternoon last, to provide for the assembling of another Convention this year. This commission consisted of the officers and one delegate from each of the States represented in the late convention. A number of the gentlemen were present, and letters from others were read, expressing their inability to attend, but their earnest desire for another Convention.

After certain preliminaries, it was resolved to assemble another Convention in Baltimore, on Thursday, the 29th of April ensuing, at 10 o'clock A.M. We learn, also, that delegates will be invited from the Municipal Corporations, Chambers of Commerce, Boards of Trade, Boards of Health, and such Medical Societies in each of the seaboard cities as may be selected by the Executive Committee.

The necessary arrangements for the call of the Convention were committed to an Executive Committee, consisting of Drs. Jewell, Hartshorne, and Biddle, of our city, and Drs. Kemp and Steiner, of Baltimore.

From the deep interest felt in this subject last year, and the great number that have since signified their appreciation of the important questions involved, and their desire to be present at the next Convention, an unusually large delegation may be expected.

The *circular* has not yet reached us.

